# Activated Stat5 levels do not increase with cell density in contrast to activated Stat3 levels in breast epithelial cell culture

**DOI:** 10.1186/1753-6561-7-S2-P31

**Published:** 2013-04-04

**Authors:** Jamaica D Cass, Mulu Geletu, Sandip SenGupta, Bruce E Elliott, Leda Raptis

**Affiliations:** 1 Division of Cancer Biology and Genetics, Queen’s University, Kingston, Canada K7L 3N6; 2 Department of Biomedical and Molecular Sciences, Queen’s University, Kingston, Canada K7L 3N6; 3 Department of Pathology and Molecular Medicine, Queen’s University, Kingston, Canada K7L 3N6

## Background

The Signal Transducer and Activator of Transcription-3 (Stat3) is activated by cytokine receptors such as the IL6 receptor (IL6R) family, as well as Src and Abl. In cancer, Stat3 can become constitutively active, regulate genes involved in survival, proliferation and invasion, and has been associated with poor survival in breast cancer patients. Our group recently discovered a novel pathway of Stat3 activation triggered by engagement of cadherins, cell to cell adhesion molecules, which induce high levels of Rac and transcriptional activation of IL6. Another Stat family member, Stat5, is known to promote breast differentiation and lactation in response to lactogenic hormones, and its activation correlates with a more differentiated breast tumour phenotype.

## Materials and method

To assess whether Stat5 activation also increases with cell density, HC11 breast epithelial cells were plated at different densities, and pY694Stat5, pY705Stat3 and corresponding target genes (cyclin D1 and p21) were assayed via Western blotting. Cyclin D1 and p21 were also stained and scored on a human breast cancer tissue microarray (n=63).

## Results

PYStat3 expression was dramatically increased in cells at higher densities, however pYStat5 levels remained constant throughout all cell densities. This indicates that Stat3 and Stat5, although activated by similar cytokines, are differentially regulated in breast development.

## Conclusions

Activated Stat5 levels do not increase with cell density in contrast to activated Stat3 levels in breast epithelial cell culture. Additionally, expression of the Stat3 target gene, cyclin D1, is correlated inversely with triple negative breast cancers, supporting a distinct role of this pathway in breast cancer subtypes. Together, our results suggest differential regulatory roles of Stat3 vs Stat5 in breast cancer.

## Financial support

CBCF, CIHR, Terry Fox Transdisciplinary Studentship in Partnership with CIHR.

